# Synergistic effects of *Bacillus* spp. and graphene oxide on nutrient cycling and soil health in peach tree rhizospheres

**DOI:** 10.3389/fmicb.2025.1712181

**Published:** 2026-02-13

**Authors:** Shuyi Chen, Bingliang Liu, Sumin Chen, Xin Yu, Jie Tang, Qiang Li

**Affiliations:** Key Laboratory of Coarse Cereal Processing, Ministry of Agriculture and Rural Affairs, Sichuan Engineering and Technology Research Center of Coarse Cereal Industrialization, School of Food and Biological Engineering, Chengdu University, Chengdu, China

**Keywords:** *Bacillus* spp., graphene oxide, microbial diversity, soil, synergy

## Abstract

**Introduction:**

Peach trees are often threatened by various diseases during cultivation.

**Methods:**

This study took the roots and rhizosphere soil of diseased peach trees as the research objects and investigated the effects of single and combined treatments of Bacillus (including *Bacillus velezensis* and *Bacillus subtilis*) and graphene oxide (GO) on peach trees.

**Results:**

The results showed that compared with the diseased control group, the single treatments of *Bacillus velezensis* (Group V) and *Bacillus subtilis* (Group S) significantly reduced soil pH (to 8.55 and 8.35, respectively) as well as the contents of calcium (Ca) and zinc (Zn), enhanced organic carbon accumulation (up to 26.43 g/kg), bringing these key indicators close to the rhizosphere levels of healthy peach trees (CK1: pH 8.42, organic carbon (OC) 24.10 g/kg). Additionally, these treatments restored nutrient cycling functions by increasing microbial diversity. The effect of GO treatment showed a concentration dependence: high-concentration GO increased the microbial Shannon index and available phosphorus content, while low-concentration GO inhibited the accumulation of available nitrogen. Among the combined treatments, the combination of *Bacillus velezensis* and GO, by synergistically regulating the contents of Ca and available nitrogen, restored rhizosphere microecological homeostasis more significantly than the combination involving *Bacillus subtilis*.

**Discussion:**

This study reveals the mechanism by which the “microbe-GO” combination achieves rhizosphere ecological remediation through the “element balance - microbial community function” linkage and provides a theoretical basis and technical strategies based on healthy microecological targets for the prevention and control of soil-borne diseases in peach trees.

## 1 Introduction

Peach trees (*Prunus persica*) are important economic fruit trees that are widely planted worldwide (Kumari et al., 2023). However, planting peach trees often faces the threat of a variety of diseases. Among them, *Colletotrichum fructicola*, a fungal disease, has attracted attention due to its fast transmission speed and severe damage. Anthracnose of peach is generally caused by *Colletotrichum acutatum* and *Colletotrichum gloeosporioides* (Karim et al., 2024). Anthracnose not only kills leaves and rots fruits, but also causes the death of the entire plant in severe cases. This severely restricts the healthy growth of peach trees (Luo et al., 2022). The occurrence of pathogenic fungi is closely associated with changes in soil microbial communities (Zhang et al., 2025; Zachow et al., 2011). The abundance and diversity of soil microorganisms are crucial for soil fertility and plant growth. For example, a long-term study has shown that adding livestock and poultry manure to fields treated with chemical fertilizers can restore bacterial diversity to levels comparable to those in unfertilized soils, mainly by regulating soil pH (Sun et al., 2015). In addition, soil microbial communities directly promote plant growth and alleviate abiotic stress in crops by improving nutrient acquisition, enhancing stress resistance, and significantly increasing the abundance of key functional microbial groups (Fadiji et al., 2026; Ben Romdhane et al., 2026). Therefore, it is important to explore effective and environmentally friendly anthracnose control strategies to ensure the safety of peach tree production.

Previous studies have used pesticides to control anthracnose, such as difenoconazole + azoxystrobin and thiophanate-methyl, to prevent and treat lucky bamboo anthracnose disease (Elshahawy and Darwesh, 2023). Propiconazole and difenoconazole are used to control anthracnose in chili fruits (Gopinath et al., 2006). In addition, some studies have used sodium diacetate to induce lipid droplet accumulation in *Colletotrichum gloeosporioides*, thereby inhibiting postharvest anthracnose in citrus (Hu et al., 2025). Studies have shown that eugenol can effectively inhibit the growth and development of the postharvest anthracnose pathogen *Colletotrichum gloeosporioides* (Tan et al., 2025). However, the widespread application of these research findings is still limited.

In recent years, novel agricultural technologies, nanomaterials, and microbial agents have shown enormous potential in the field of plant disease prevention and control (Negi et al., 2024; Zhang et al., 2024). As a typical 2D nanomaterial, graphene oxide (GO) has excellent antibacterial properties, good biocompatibility, and environmental stability (Li et al., 2026; Lata et al., 2025). It affects the growth of microbial communities (Ahamed et al., 2024; Yan et al., 2022). Nevertheless, there is a lack of systematic studies on the use of GO in managing peach anthracnose and its impact on the microecological environment in the rhizosphere of peach trees. In addition, the use of microbial fluids with antimicrobial activity to mitigate the effects of diseases on crops is increasingly favoured in agriculture as an environmentally friendly strategy (Díez-Méndez et al., 2025). Beneficial microorganisms such as *Bacillus subtilis*, *Candida utilis*, and *Pseudomonas lactis* can inhibit the growth of pathogenic bacteria through mechanisms including nutrient competition, antibiotic production, or adsorbing and degrading toxins (Wang et al., 2023). Moreover, genetically modified microbial preparations can better control plant pathogens, promote plant growth, and improve crop stress resistance (Chen et al., 2022). Especially for peach anthracnose, screening and applying highly effective antagonistic microbial resources are important ways to reduce the use of chemical pesticides and achieve sustainable agriculture.

Members of the genus *Bacillus*, particularly *B. velezensis* and *B. subtilis*, have received widespread attention due to their dual roles in biocontrol and promoting plant health (Chen et al., 2025; Etesami et al., 2023). Both of these strains are capable of producing a variety of secondary metabolites with broad-spectrum antibacterial activity, showing strong inhibitory effects against multiple plant pathogens (Liang et al., 2024; Chen et al., 2025). In addition, they can promote plant growth, enhance stress resistance, and contribute to increased crop yields (Kang et al., 2025; Hashem et al., 2019). Studies have shown that *B. subtilis* can inhibit pathogens by producing volatile organic compounds that suppress the expression of virulence genes (Liu et al., 2025). While *B. velezensis* is also renowned for its powerful biocontrol efficacy as well as its ability to degrade organic pollutants and increase soil organic carbon (Zhong et al., 2024). However, the aforementioned studies mostly focus on the role of a single material within a single system. When *Bacillus* sp. and GO are applied together to the rhizosphere of diseased fruit trees, the interaction between them remains unclear. Can this interaction drive the restoration of an imbalanced disease-associated micro-ecosystem to a healthy state by synergistically regulating the function of the rhizosphere microbial community and the soil chemical environment? Its specific mechanism is still unclear. This study investigates the effects of graphene oxide and *Bacillus velezensis* and *Bacillus subtilis* microbial agents on controlling peach anthracnose, and for the first time combines *Bacillus* with GO. The effects of the treatments on the physicochemical properties and microbial community structure of peach rhizosphere soil were further analyzed. We hypothesize that the combined treatment of GO and microbial agents can improve soil physicochemical properties and enhance the diversity of rhizosphere microbial communities. This study provides a scientific basis for the green prevention and control of peach anthracnose and contributes to the application of nanomaterials and microbial agents in sustainable agriculture.

## 2 Materials and methods

### 2.1 Experimental protocol

The graphene oxide (GO) utilized in this research was bought from Shanghai Yuanye Biotechnology Co., Ltd. The bacteria used in the experiment were *B. velezensis* and *B. subtilis* originating from the Microbiology Conservation Centre of Chengdu University. These two bacteria are often reported to have bacteriostatic properties. The experimental location was in the Longquanshan area, Sichuan Province, China. We applied the liquid agent twice to diseased peach trees in May 2024 and performed rhizosphere soil sampling and plant root collection in July 2024 (Aimed at assessing the immediate effects of various treatments on rhizosphere health during peak crop demand periods.). When sampling, the top 0 to 5 cm of soil was removed, plant roots were shaken to get rid of loose soil, and the remaining soil collected from the roots with a sterile brush was considered rhizosphere soil.

In the experiment, the solutions were applied to the roots of diseased peach trees as follows: (1) V: *B. velezensis* 300 mL, (2) S: *B. subtilis* 300 mL, (3) L: 250 mg/L GO 300 mL, (4) H: 300 mL of 1,000 mg/L GO, (5) VL: 150 mL each of *B. velezensis* and 250 mg/L GO, (6) VH: 150 mL each of *B. velezensis* and 1,000 mg/L GO, (7) SL: 150 mL each of *B. subtilis* and 250 mg/L GO, (8) SH: 150 mL each of *B. subtilis* and 1,000 mg/L GO. Two bacterial solutions were fermented in LB liquid media at 30 °C for 24 h. The control groups for the experiment were as follows: CK1: a blank control group without diseased peach trees, CK2: a blank control group with diseased peach trees.

### 2.2 Soil physicochemical measurements

Soil pH was determined by the glass electrode method. Soil organic carbon was measured using the potassium dichromate oxidation-external heating method (Shen et al., 2024). Soil total nitrogen was determined by the Kjeldahl method. Soil total phosphorus and available phosphorus were analyzed by the molybdenum-antimony anti-spectrophotometric method (Zhang et al., 2024). Soil total potassium and available potassium were detected by the flame photometric method (Wang et al., 2024). Soil alkaline hydrolyzable nitrogen was determined by the alkaline hydrolysis diffusion method (Fan et al., 2025). The multi-element content in soil was measured by the tetra-acid digestion-inductively coupled plasma mass spectrometry method (Oliveira et al., 2024).

### 2.3 DNA extraction and PCR amplification

Genomic DNA of endophytic bacteria in roots, rhizosphere soil bacteria, and fungi was extracted using the SDS method (Thermo Fisher Scientific, China) (Three tests are performed for each sample) (Tanase et al., 2015). The diluted DNA was used as a template to amplify the V4 hypervariable region of the bacterial 16S rRNA gene via the primers 515F (5’-GTGCAGCCGCGGTAA-3′) and 806R (5’-GGACTACHVGGGTWTCTAAT-3′). The fungal ITS fragment was amplified via the primers ITS5-1737F (5’-GGAAGTAAAGTCGTAACAAGG-3′) and ITS2-2043R (5’-GCTGCGTCTTCATCGATGC-3′). The NEBNext® Ultra™ II DNA Library Prep Kit was used to construct the library. After library construction, Qubit quantification and Q-PCR quantification analysis were performed on the library, respectively (Bokulich et al., 2018). Once the library passed quality inspection, on-machine sequencing was carried out using the NovaSeq6000 sequencing platform (Bolyen et al., 2019).

### 2.4 Analysis of 16S rRNA and ITS sequencing data

The data for each sample was separated from the offloading data using the barcode sequence and the PCR amplification primer sequences. FLASH was used after truncating the barcode and primer sequences (Magoč and Salzberg, 2011). The reads were obtained by splicing the raw tags of each sample. After strict filtering with fastp software, clean tags were obtained from the raw tags (Bokulich et al., 2013). In order to remove chimeric sequences and obtain the final effective tags, the clean tags were compared with the species annotation database (Silva database,^1^ for 16S/18S, Unitedatabase,^2^ for ITS) (Edgar et al., 2011; Wang et al., 2021). The DADA2 (3.14) module in the QIIME2 (2024.05) software was used for noise reduction to obtain the final ASVs (Bolyen et al., 2019). Species annotation was performed for each ASV representative sequence, and a multiple sequence alignment was performed to construct a phylogenetic tree.

### 2.5 Identification and whole genome sequencing analysis of *Bacillus* spp. strains

Two *Bacillus* spp. strains were identified using 16S rRNA gene sequencing. Total DNA was extracted using the GenElute Bacterial Genomic DNA Kit (Merck, Germany). The DNA extract was amplified with primers 27F (5’-GAGAGTTTGATCCTGGCTCAG-3′) and 1492R (5’-TACGGCTACCTTGTTACGAC-3′), followed by sequencing using the Sanger method on an ABI 3730XL DNA Analyzer (Applied Biosystems, USA). To identify the target strain, the obtained sequences were input into the NCBI database for a BLAST search, and similar sequence comparison results were obtained. Finally, a phylogenetic tree was constructed using MEGA11 software.

Genomic DNA was extracted via the SDS method. After sample detection and purification, a 1D library was constructed according to the instructions using the SQK-LSK110 Kit (Oxford Nanopore Technologies, UK), while a small-fragment library was built with the VAHTS® Universal Plus DNA Library Prep Kit (Vazyme, China). After passing quality inspection, the libraries were sequenced using the Nanopore PromethION and Illumina NovaSeq 6000 platforms, respectively. Following sequencing, quality control was performed on the raw data; low-quality and short-length reads were filtered out before *de novo* assembly, and error correction was conducted on the assembled draft genome. Subsequent steps included genomic structure analysis and functional annotation. The R package circlize (Gu et al., 2013) was used to integrate predicted genomic information (e.g., genomic structure annotation, GC distribution, and Annotation of Orthologous Gene Clusters functional annotation) and generate a genomic circular map (Liu et al., 2025).

### 2.6 Data analysis

Before statistical analysis, a normality test was performed on all response variables. Statistical analysis was performed using SPSS 23.0. One-way analysis of variance (ANOVA) and *post-hoc* least significant difference (LSD) tests were used to evaluate the effects of different treatments on plant and soil factors. Microsoft Excel 2019 was used to calculate the means and standard deviations. Origin 2021 software was used for drawing the figures. The alpha diversity indices of different samples were calculated via QIIME2 software. Beta diversity index was used to examine differences among groups, and multivariate statistical methods were conducted through Principal Co-ordinates Analysis (PCoA). The microbial community composition of various samples was comparatively analyzed using the nonmetric multidimensional scaling (NMDS) method (NMDS reflects the species information contained in samples as points on a two-dimensional plane. It can overcome the drawbacks of linear models (PCoA) and better represent the nonlinear structure of ecological data.). To make functional predictions of different microorganisms, the microbial communities in the ecological samples were used to perform functional prediction analysis via PICRUSt2 (V2.3.0) and FunGuild (V0.3.1) (Douglas et al., 2020; Nguyen et al., 2016). The main variables affecting the diversity of bacteria and fungi were analyzed via Pearson correlation.

## 3 Results

### 3.1 Changes in soil physicochemical properties

The physicochemical characteristics of the rhizosphere soils in the distinct groups are displayed in Table 1, showing weakly alkaline pH values for all samples except for CK2. The pH value of the SH group was the lowest at 8.1, which was relatively close to neutral. Compared with the CK2 treatment, the V treatment significantly decreased the soil pH by 10.84%. Among all the groups, the V group had the highest OC content at 26.43. The Ca content in the CK2 group was significantly greater than that in the other groups, reaching 42.17 g/kg, whereas the Ca contents in the V, S, L, and SL groups were low, at less than 13 g/kg (*p* < 0.05). The Zn content in CK2 was significantly greater than that in the other groups (*p* < 0.05), while the differences in the contents of Fe and Mg among the groups were relatively small. The soil’s total nitrogen (TN), total potassium (TK), available potassium (AK), total phosphorus (TP), and available phosphorus (AP) indicators in CK2 were significantly greater than those in the other groups (*p* < 0.05). Compared with the CK2 treatment, the V treatment significantly decreased the soil pH, available nitrogen (AN), AP, and AK by 10.84, 32.90, 80.17, and 51.43%, respectively, and the values were closer to those of the CK1 treatment (*p* < 0.05). The TN and AN contents were the lowest in Group L.

**Table 1:** Physicochemical properties of the rhizosphere soil.

Groups	CK1	CK2	V	S	L	H	VL	VH	SL	SH
pH	8.42 ± 0.05d	9.59 ± 0.02a	8.55 ± 0.06bc	8.35 ± 0.03ef	8.51 ± 0.02c	8.58 ± 0.01b	8.40 ± 0.03de	8.25 ± 0.04 g	8.31 ± 0.02f	8.10 ± 0.03 h
OC(g/kg)	24.10 ± 0.29c	25.82 ± 0.47b	26.43 ± 0.48a	12.72 ± 0.22d	24.26 ± 0.55c	12.78 ± 0.22d	10.96 ± 0.13e	11.06 ± 0.22e	10.51 ± 0.29e	12.73 ± 0.25d
Ca(g/kg)	21.33 ± 1.10c	42.17 ± 2.08a	11.97 ± 0.45e	12.93 ± 0.64e	11.73 ± 0.40e	17.17 ± 0.85d	23.90 ± 0.53b	23.43 ± 0.31b	12.83 ± 0.31e	17.80 ± 0.87d
Fe(g/kg)	43.43 ± 1.37a	41.57 ± 0.92ab	38.37 ± 1.21 cd	38.37 ± 1.27 cd	40.27 ± 1.17bc	36.43 ± 1.15d	37.17 ± 1.38d	37.13 ± 0.75d	37.30 ± 0.61d	38.10 ± 1.23d
Mg(g/kg)	15.70 ± 0.17a	15.90 ± 0.66a	13.30 ± 0.56 cd	13.73 ± 0.50bcd	14.43 ± 0.47b	13.37 ± 0.45 cd	13.10 ± 0.52d	13.37 ± 0.32 cd	13.53 ± 0.15 cd	14.07 ± 0.38bc
Zn(mg/kg)	96.67 ± 4.07b	195.83 ± 4.92a	79.80 ± 1.21 cd	76.83 ± 2.67de	75.20 ± 2.59de	76.67 ± 2.42de	72.17 ± 2.82e	80.80 ± 2.26 cd	72.63 ± 1.07e	84.97 ± 4.20c
TN(g/kg)	1.80 ± 0.05c	1.98 ± 0.05a	1.65 ± 0.01de	1.81 ± 0.05c	1.44 ± 0.02f	1.85 ± 0.06bc	1.70 ± 0.02d	1.63 ± 0.03e	1.80 ± 0.05c	1.91 ± 0.01b
TP(g/kg)	1.53 ± 0.02b	2.21 ± 0.01a	1.02 ± 0.01e	0.98 ± 0.01f	0.75 ± 0.02i	0.79 ± 0.01 h	1.07 ± 0.02d	1.55 ± 0.02b	0.94 ± 0.02 g	1.38 ± 0.02c
TK(g/kg)	22.27 ± 0.25b	24.77 ± 0.12a	18.70 ± 0.26d	19.60 ± 0.26c	18.57 ± 0.15d	17.80 ± 0.26e	18.40 ± 0.26d	18.73 ± 0.15d	18.40 ± 0.26d	19.33 ± 0.21c
AN(mg/kg)	116.67 ± 1.53f	181.33 ± 6.11a	121.67 ± 5.13ef	143.00 ± 5.00b	103.00 ± 6.08 g	129.67 ± 2.08 cd	137.67 ± 2.31b	135.67 ± 3.51bc	126.67 ± 4.92de	179.33 ± 3.21a
AP(mg/kg)	22.73 ± 1.00ef	96.67 ± 1.60a	19.17 ± 0.50 g	24.17 ± 0.91de	15.43 ± 0.35 h	22.67 ± 0.64ef	25.37 ± 0.91d	49.40 ± 1.44b	21.83 ± 0.96f	47.07 ± 0.93c
AK(mg/kg)	302.33 ± 5.86d	597.67 ± 37.17a	292.00 ± 7.55d	352.67 ± 1.53c	198.67 ± 2.89f	230.67 ± 0.58e	227.00 ± 2.65e	356.67 ± 2.52c	227.67 ± 3.06e	517.33 ± 13.80b

Letters in different columns in the same row indicate significant differences (*P* < 0.05). V: *B. velezensis* 300 ml, S: *B. subtilis* 300 ml, L: 250 mg/L GO 300 mL, H: 300 mL of 1,000 mg/L GO, VL: 150 ml each of *B. velezensis* and 250 mg/L GO, VH: 150 mL each of*B. velezensis* and 1,000 mg/L GO, SL: 150 mL each of *B. subtilis* and 250 mg/L GO, SH: 150 mL each of *B. subtilis* and 1,000 mg/L GO.

### 3.2 Community composition of microorganisms

High-throughput sequencing was used to analyze the samples treated in different ways. Supplementary Figure S1 displays the OTU dilution curve. When the sequencing read lengths reached above 20,000, the rarefaction curve analysis indicated a progressive stabilization of the dilution curve, guaranteeing sufficient sequencing depth for all samples and upholding the accuracy and reliability of microbial diversity analysis. At the identity thresholds of 97.00% and 7,814 ASVs for endophytic bacteria (Supplementary Figure S2A), 11,501 ASVs for rhizosphere soil bacteria and 8,725 ASVs for fungi were identified (Supplementary Figures S2B, C). Among the endophytic bacteria, there are a total of 121 ASVs in all the overlapping groups, of which the S, H, and SL groups had 1,273, 1,305, and 1,035 ASVs respectively, accounting for 46.24% of the total number of ASVs in all groups. Among the rhizosphere soil bacteria, there were 701 ASVs in all the overlapping groups, including 1,474 ASVs in the SL group and 735 ASVs in the CK2 group. Among the rhizosphere fungi, the number of ASVs in CK1 was the lowest (613), the maximum number in the S group was 1,058. There are 109 ASVs in the overlapping region of each group.

**Figure 1:**
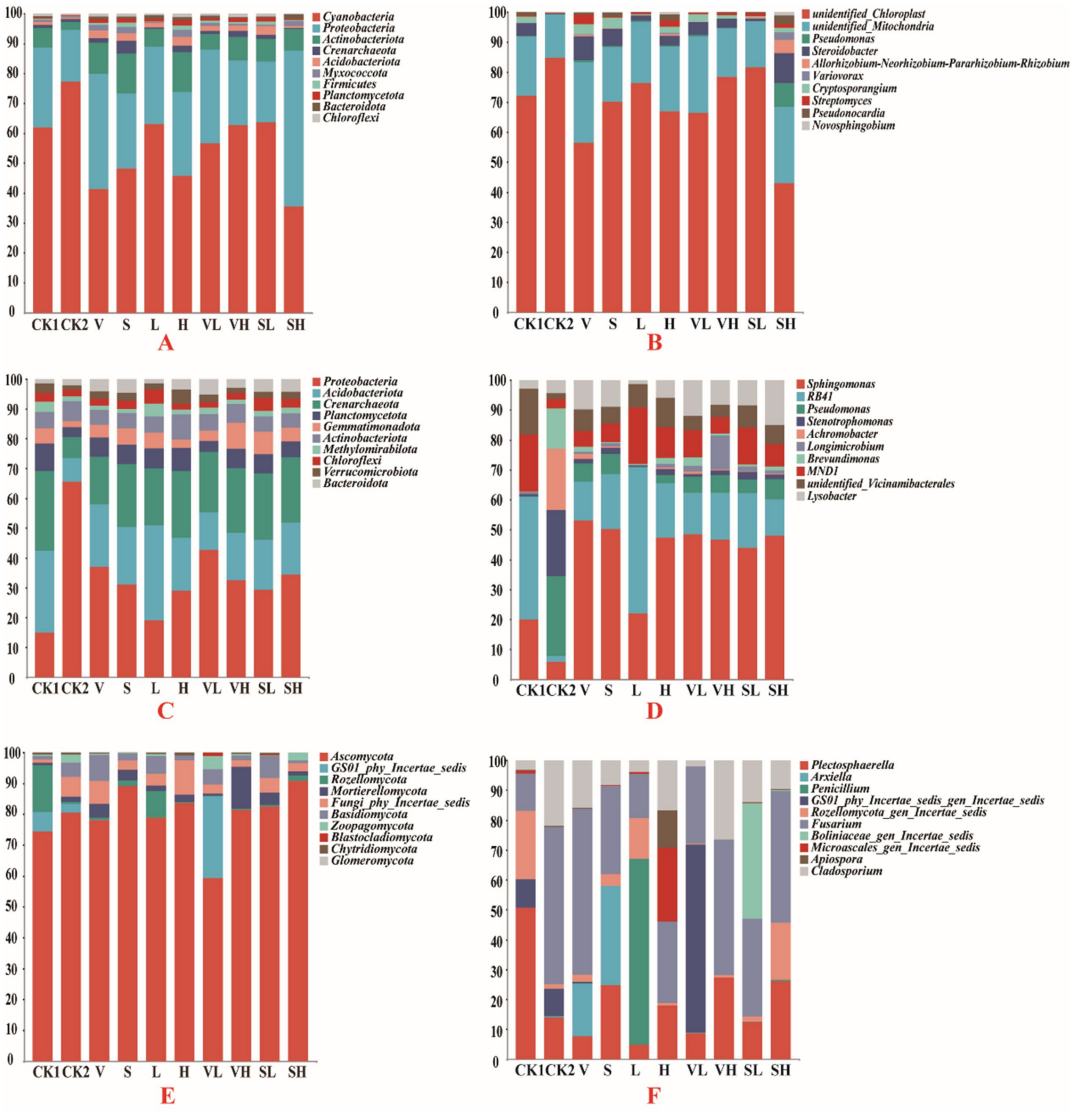
Relative abundance of the top 10 bacterial and fungal taxa in the root and rhizosphere soils at the phylum and genus levels. **(A)** Root endophyte community at the phylum level, **(B)** Rhizosphere soil bacterial community at the phylum level, **(C)** Rhizosphere soil fungal community at the phylum level, **(D)** Root endophyte community at the genus level, **(E)** Rhizosphere soil bacterial community at the genus level, **(F)** Rhizosphere soil fungal community at the genus level. V: *B. velezensis* 300 mL, S: *B. subtilis* 300 mL, L: 250 mg/L GO 300 mL, H: 300 mL of 1,000 mg/L GO, VL: 150 mL each of *B. velezensis* and 250 mg/L GO, VH: 150 mL each of *B. velezensis* and 1,000 mg/L GO, SL: 150 mL each of *B. subtilis* and 250 mg/L GO, SH: 150 mL each of *B. subtilis* and 1,000 mg/L GO.

At the phylum level, Cyanobacteria, Proteobacteria, and Actinobacteria dominated the endophytic bacteria (Figure 1A). Cyanobacteria was the most abundant bacterial phylum, accounting for 54.93% of the endophytic bacterial communities. The soil bacterial communities included mainly Proteobacteria, Acidobacteria, and Crenarchaeota, which accounted for 31.43, 17.53, and 18.48% of the total communities, respectively (Figure 1C). Compared with the CK1 group, the abundance of Proteobacteria in the CK2 group was the lowest, and the abundance of Proteobacteria in the remaining treatment groups increased. Among the soil fungi, Ascomycota accounted for the greatest percentage (57.57%), followed by Fungi_phy_Incertae_sedis (3.19%), GS01_phy_Incertae_sedis (2.81%), and Basidiomycota (2.71%) (Figure 1E).

At the family level, *mitochondria* accounted for 15.95% (Supplementary Figure S3A), *Steroidobacteraceae* accounted for 3.84%, *Nitrososphaeraceae* accounted for 1.48%, and *Cryptosporangiaceae* accounted for 1.29%. In the soil bacterial communities (Supplementary Figure 3B), *Nitrososphaeraceae* accounted for the greatest proportion (18.46%), followed by *Sphingomonadaceae* (7.53%) and *Vicinamibacteraceae* (5.22%), with the lowest abundance of *Nitrososphaeraceae* and *Vicinamibacteraceae* (5.22%) in the CK2 group. In the soil fungal communities (Supplementary Figure 3C), *Plectosphaeraceae* had the highest abundance (13.94%), followed by *Nectriaceae* (13.62%) and *Aspergillaceae* (3.33%).

At the genus level, *unidentified_Chloroplast* and *unidentified_Mitochondria* accounted for the majority of the endophytic bacteria, accounting for 54.83 and 15.95%, respectively (Figure 1B), followed by *Steroidobacter* (3.17%) and *Cryptosporangium* (1.29%). *Sphingomonas* (5.15%), RB41 (2.52%), and *Pseudomonas* (1.26%) accounted for more of the soil bacterial community (Figure 1D). In particular, the CK2 group presented the lowest abundance of *Sphingomonas* and Rb41. Compared with that in CK1, the abundance of *Sphingomonas* increased in all the treatment groups. At the genus level, the abundance of some fungi varied across treatment groups, with *Plectosphaerella* being the most abundant genus in CK1, *Fusarium* the most abundant genus in CK2, and *Penicillium* the most abundant genus in group L (Figure 1F).

### 3.3 Microbial community diversity

**Figure 2:**
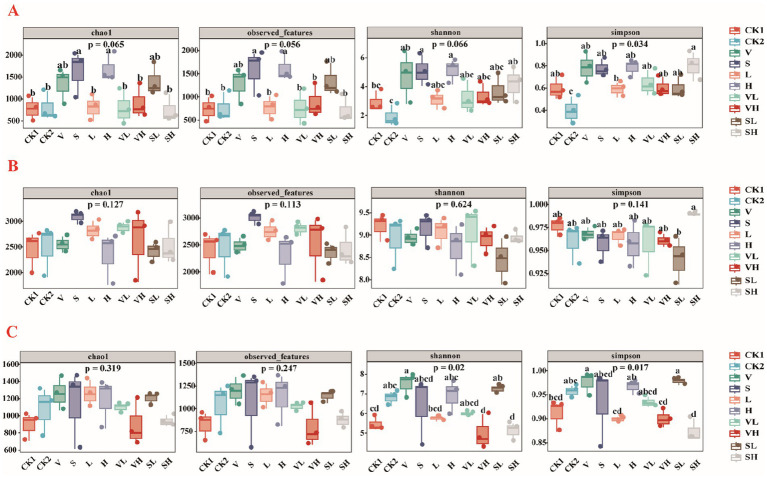
Box line plots of the microbial diversity and richness of root endophytes **(A)**, rhizosphere soil bacteria **(B)**, and rhizosphere soil fungi **(C)** in different samples [The letters above the box plot indicate the significance of differences between groups. The same letter means there is no significant difference between groups (*p* > 0.05), while different letters indicate a significant difference between groups (*p* < 0.05)] V: *B. velezensis* 300 mL, S: *B. subtilis* 300 mL, L: 250 mg/L GO 300 mL, H: 300 mL of 1,000 mg/L GO, VL: 150 mL each of *B. velezensis* and 250 mg/L GO, VH: 150 mL each of *B. velezensis* and 1,000 mg/L GO, SL: 150 mL each of *B. subtilis* and 250 mg/L GO, SH: 150 mL each of *B. subtilis* and 1,000 mg/L GO.

Utilizing the Chao1, observed_features, Shannon, and Simpson indices, the alpha diversity of the species was characterized. Among the endophytic bacteria (Figure 2A), the Shannon and Simpson indices of the V, S, and H groups were greater than those of the other treatment groups, whereas the CK2 group presented the lowest Shannon index and Simpson index values. The Simpson index of the SH group was significantly greater than that of the CK2 and L groups (*p* < 0.05). For the soil bacteria, there was no significant difference in the alpha diversity index among the treatments (Figure 2B), with the Chao1 index of the S group being greater and the Simpson index of the SH group being greater. For the soil fungi (Figure 2C), the Shannon index of Group V was significantly higher than that of group VH (*p* < 0.05). The Shannon index of the SL group was significantly greater than that of the SH group. The Simpson indices of the V and SL groups were the highest and were significantly greater than those of the CK1, L, VH, and SH groups (*p* < 0.05).

**Figure 3:**
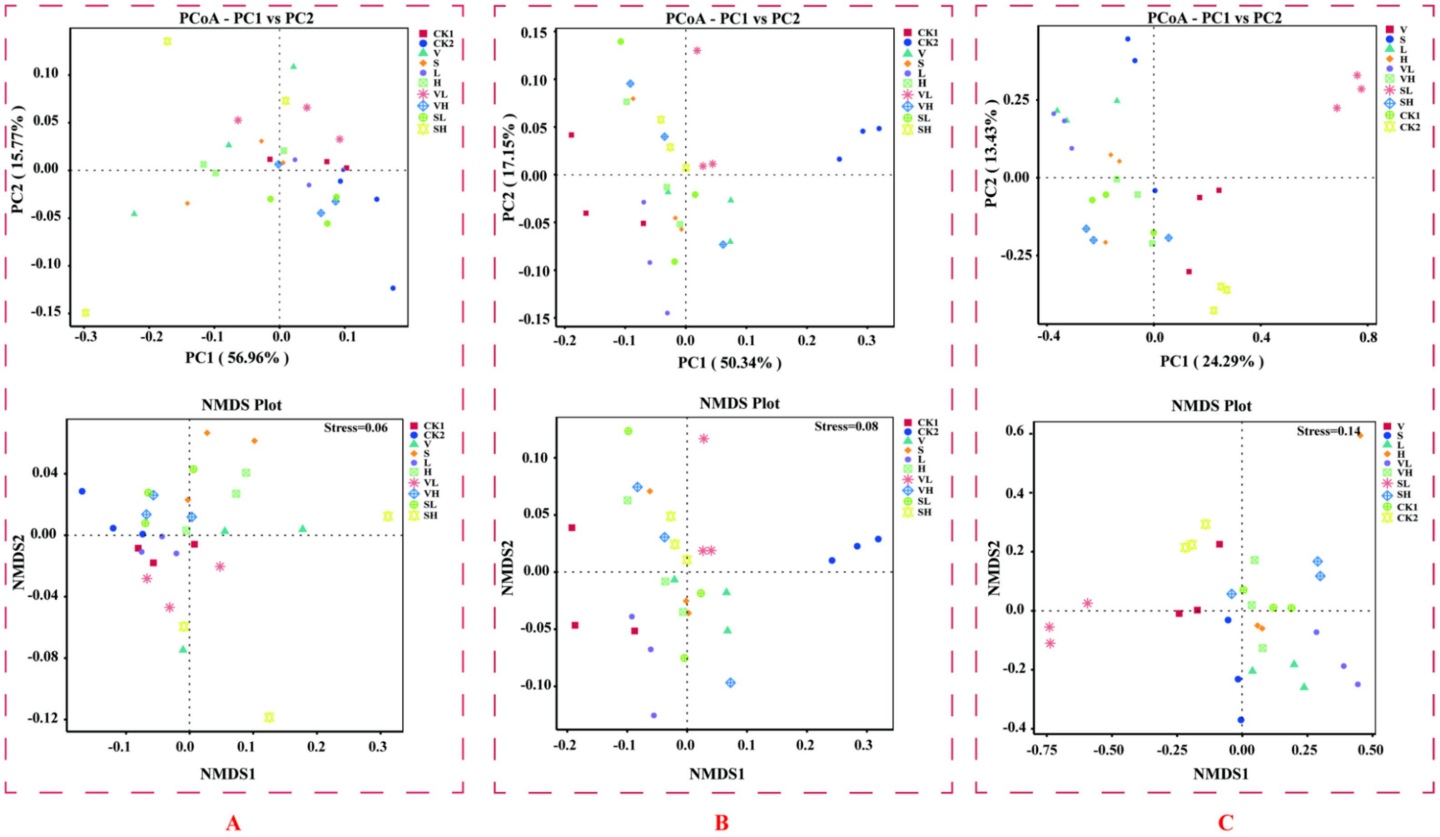
ASV-based PCoA and unweighted Unifrac-based NMDS analysis. **(A)** Root endophytes, **(B)** rhizosphere soil bacteria, and **(C)** rhizosphere soil fungi (In a PCoA plot, the closer the samples are, the more similar their species composition structure is. In an NMDS plot, the degree of difference between different samples is reflected by the distance between the points). V: *B. velezensis* 300 mL, S: *B. subtilis* 300 mL, L: 250 mg/L GO 300 mL, H: 300 mL of 1,000 mg/L GO, VL: 150 mL each of *B. velezensis* and 250 mg/L GO, VH: 150 mL each of *B. velezensis* and 1,000 mg/L GO, SL: 150 mL each of *B. subtilis* and 250 mg/L GO, SH: 150 mL each of *B. subtilis* and 1,000 mg/L GO.

We investigated the beta diversity among microbial communities from different regions through PCoA analysis at the genus level and NMDS analysis based on Unweighted Unifrac distance. For endophytic bacteria (Figure 3A), PCoA revealed that PC1 and PC2 explained 56.96 and 15.77% of the variance, respectively. Except for Group V and Group SH, the community similarity between groups was high, and the NMDS stress value was 0.06. For soil bacteria (Figure 3B), PCoA revealed that PC1 and PC2 explained 50.34 and 17.15% of the variance, respectively. There was partial overlap between Group V, Group S, and Group H, and the NMDS stress value was 0.08. For soil fungi (Figure 3C), PCoA revealed that PC1 and PC2 explained 24.29 and 13.43% of the variance, respectively. The samples in the SL group were relatively clustered, but the distance to the other groups was relatively large, and the NMDS stress value was 0.14.

### 3.4 Correlation analysis

**Figure 4:**
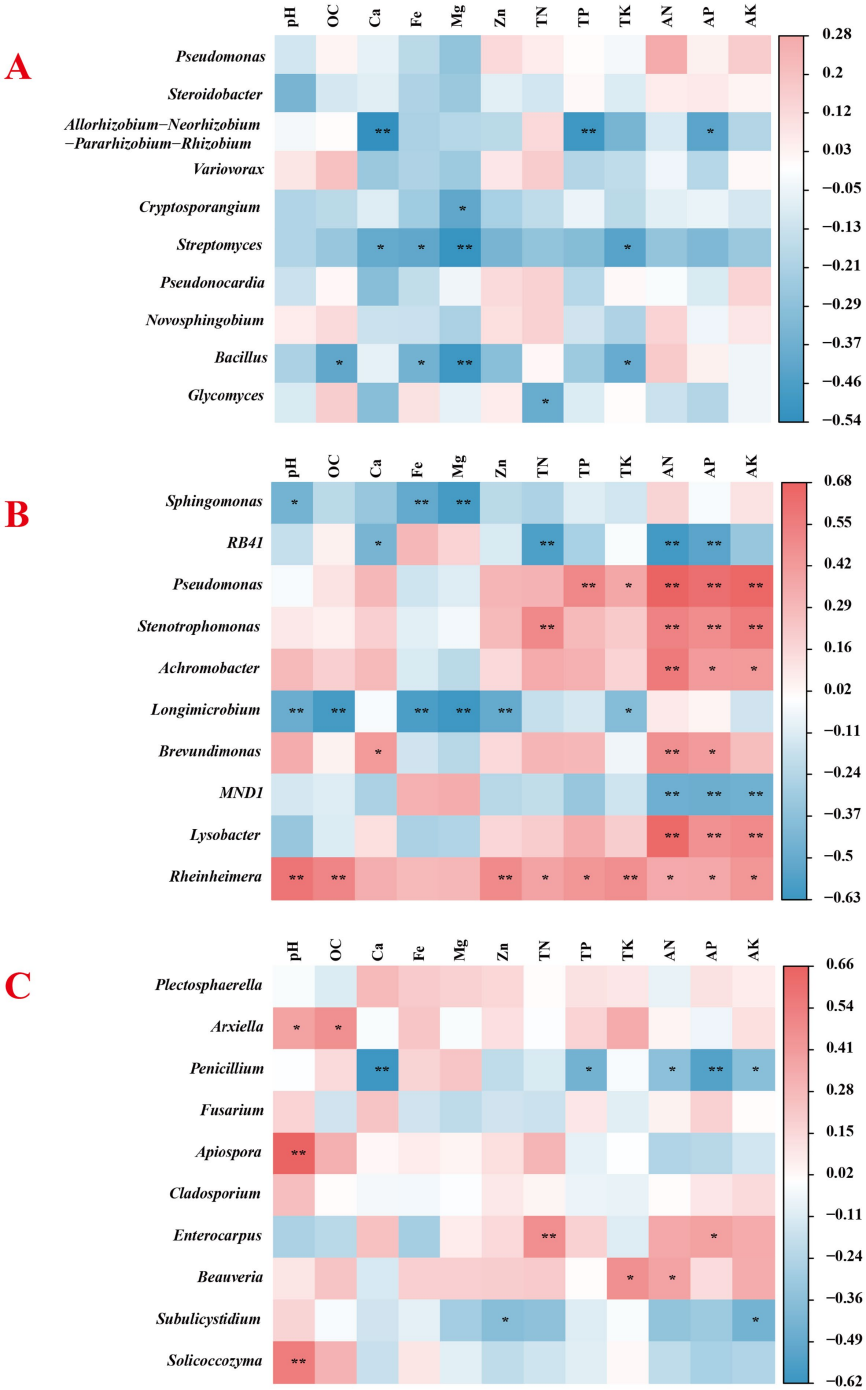
Heatmap of correlations between dominant genes of root endophytes **(A)**, rhizosphere soil bacteria **(B)**, and rhizosphere soil fungi **(C)** and soil physicochemical factors (*p* < 0.05).

The relationship between soil physicochemical parameters and dominant species at the genus level of microbial communities was examined through Pearson correlation analysis (The functional traits of microorganisms are generally more conserved at the genus level than at other levels, making inferences based on taxonomic units more functionally meaningful. Additionally, genus-level taxonomic units often respond more sensitively and specifically to environmental gradients (such as pH and nutrient availability) than higher-level taxa.). The results revealed that the bacterial structure in the root system was significantly negatively correlated with Ca, Mg, and TP (Figure 4A). Soil bacteria *Pseudomonas*, *Stenotrophomonas*, *Achromobacter*, *Lysobacter*, and *Rheinheimera* were significantly positively correlated with AN, AP, and AK, while *Longimicrobium* was significantly negatively correlated with soil pH, OC, Fe, and Mg (Figure 4B). The soil fungus *Penicillium* was significantly negatively correlated with Ca, TP, AN, AP, and AK, and *Arxiella*, *Apiospora*, and *Solicoccozyma* were significantly positively correlated with soil pH (Figure 4C).

### 3.5 Functional enrichment of microbial communities

**Figure 5:**
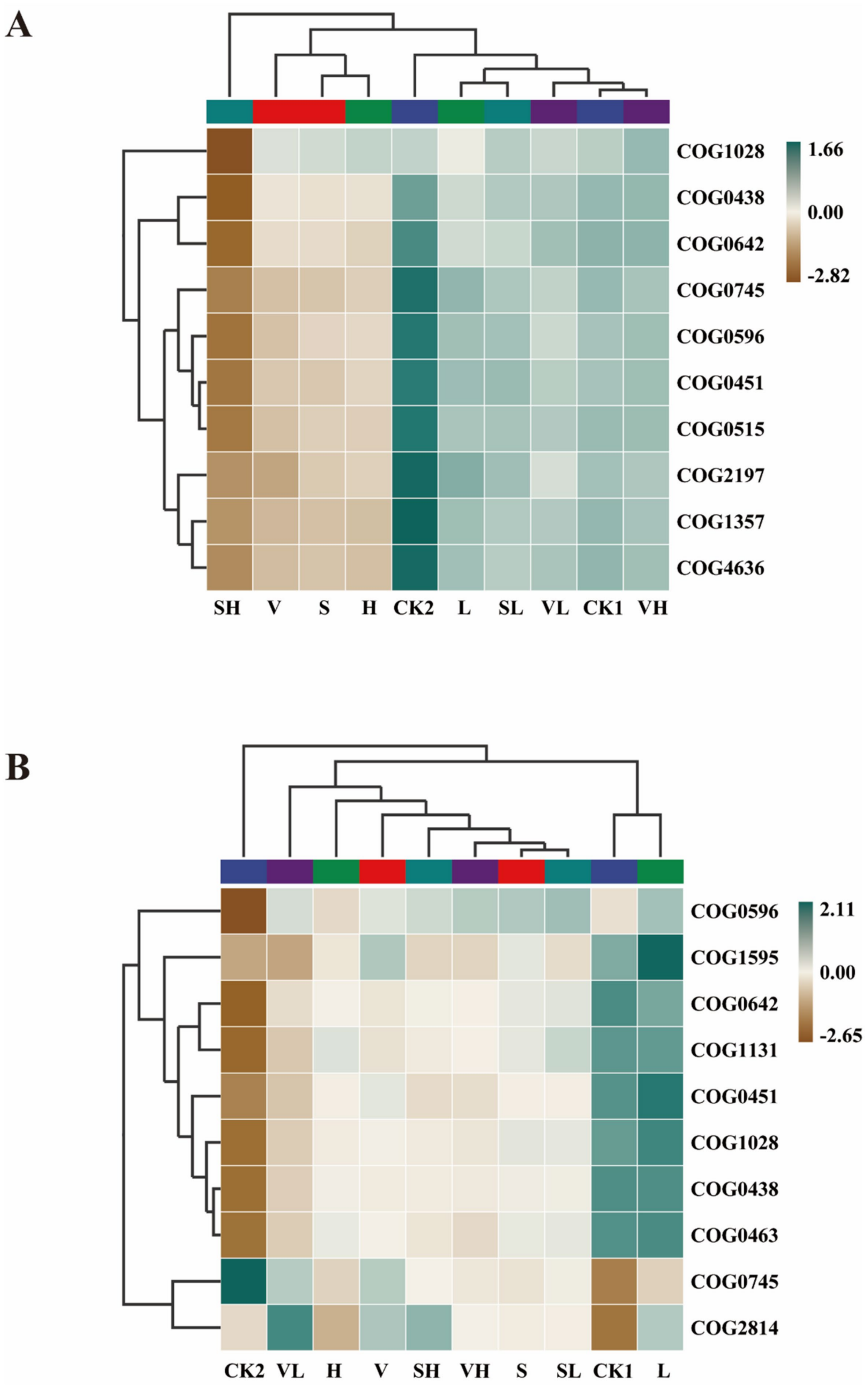
Heatmap of PICRUSt2 functionally annotated clusters of root endophytes **(A)** and rhizosphere soil bacteria **(B)**. V: *B. velezensis* 300 mL, S: *B. subtilis* 300 mL, L: 250 mg/L GO 300 mL, H: 300 mL of 1,000 mg/L GO, VL: 150 mL each of *B. velezensis* and 250 mg/L GO, VH: 150 mL each of *B. velezensis* and 1,000 mg/L GO, SL: 150 mL each of *B. subtilis* and 250 mg/L GO, SH: 150 mL each of *B. subtilis* and 1,000 mg/L GO.

PICRUSt2 was employed for predicting bacteria functions, with the COG reference database being used. (Based on all high-quality ASVs obtained from 16S rRNA gene sequencing analysis.) These pathways mainly belong to categories such as energy metabolism, amino acid metabolism, and secondary metabolite biosynthesis, and they are directly related to soil nutrient transformation and rhizosphere health. Fungal functions were predicted through FUNGuild, and the fungal taxa were annotated as ecological functional groups. Among the endophytic bacteria in the root system (Figure 5A), the functions of endonucleases, the Uma2 family (COG4636), and the DNA-binding response regulator (COG2197) were greater in the CK2 group than in the CK1 group. The function of DNA-binding response regulator (COG2197) was increased in Group V. The functions of NAD(P)-dependent dehydrogenase (COG1028) and glycosyltransferase involved in cell wall biosynthesis (COG0438) were weakened in the SH group. In soil bacteria (Figure 5B), glycosyltransferases involved in cell wall biosynthesis (COG0463), nucleoside-diphosphate-sugar epimerases (COG0451), etc., have strong functions, such as DNA-binding response regulators (COG0745) and predicted arabinose efflux permeases (COG2814). Weak function. Compared with CK1, CK2 has increased DNA-binding response regulator (COG0745) and reduced functions of pimeloyl-ACP methyl ester carboxylesterase (COG0596) and signal transduction histidine kinase (COG0642). The function of the predicted arabinose efflux permease (COG2814) was enhanced in the VL group, and the function of the DNA-directed RNA polymerase specialized sigma subunit (COG1595) was enhanced in the L group.

**Figure 6:**
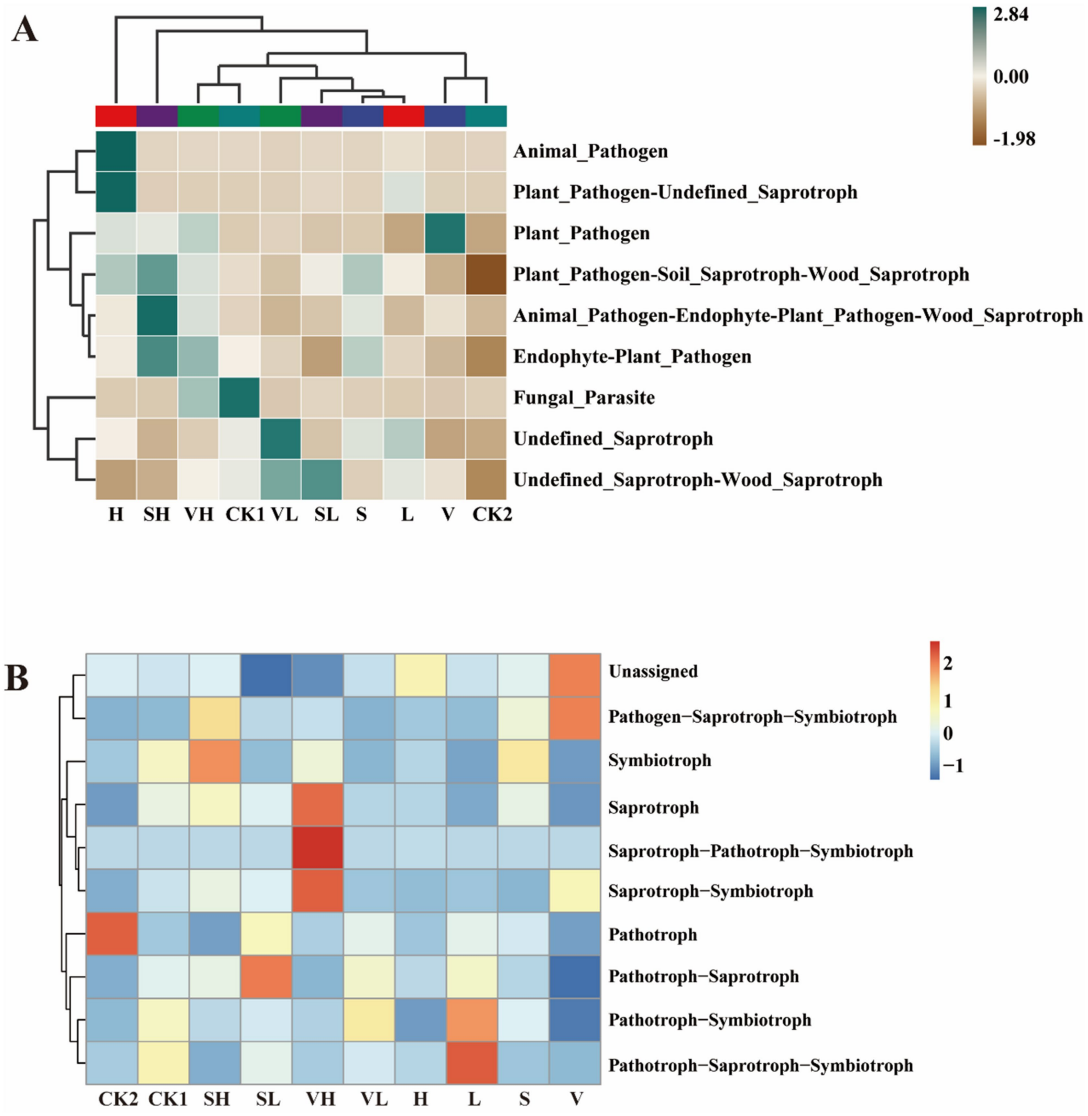
Heatmap of guild **(A)** and mode **(B)** annotated clustering of FunGuild functions of rhizosphere soil fungi. V: *B. velezensis* 300 mL, S: *B. subtilis* 300 mL, L: 250 mg/L GO 300 mL, H: 300 mL of 1,000 mg/L GO, VL: 150 mL each of *B. velezensis* and 250 mg/L GO, VH: 150 mL each of *B. velezensis* and 1,000 mg/L GO, SL: 150 mL each of *B. subtilis* and 250 mg/L GO, SH: 150 mL each of *B. subtilis* and 1,000 mg/L GO.

According to the function of FunGuild, the functions of the soil fungi in the samples could be divided into 35 categories (Figure 6A), of which Unassigned accounted for the largest proportion (60.46%), followed by other guilds such as Undefined Saprotroph (11.75%), Plant Pathogen-Soil Saprotroph-Wood Saprotroph (8.62%), and Plant Pathogen (8.26%). Compared with that in CK1, the abundance of Fungal Parasite was lower in all groups, the abundance of Plant Pathogen-Soil Saprotroph-Wood Saprotroph was significantly lower in CK2, the abundance of Plant Pathogen was significantly greater in Group V, the abundances of Animal Pathogen and Plant Pathogen-Undefined Saprotroph were significantly greater in Group H, the abundance of Undefined Saprotroph was significantly greater in the SH group, and the abundance of Animal Pathogen-Endophyte-Plant Pathogen-Wood_Saprotroph was significantly greater in the SH group (*p* < 0.05). Compared with CK2, the abundance of Animal Pathogen and Plant Pathogen-Undefined Saprotroph in group H increased significantly, the abundance of Undefined Saprotroph in group VL increased significantly, and the abundance of Animal Pathogen-Endophyte-Plant Pathogen-Wood Saprotroph in group SH increased significantly (*p* < 0.05). The trophic modes of all soil samples were categorized into 10 types (Figure 6B). The Unassigned trophic mode accounted for the largest proportion (60.24%), with the Saprotroph (14.93%), Pathotroph-Saprotroph (11.31%), Pathotroph (8.65%), and Pathotroph-Symbiotroph (3.50%) modes following behind. Compared with those in the CK1 group, the fungi in the CK2 group were pathotrophic, the fungi in the L group were pathotrophic−Saprotrophic−Symbiotic, and those in the VH group were mainly saprotrophic−pathotrophic−symbiotic.

### 3.6 Identification and whole genome sequencing of *Bacillus* spp

16S rRNA sequencing was conducted on the two bacteria utilized in the study, and their sequences were compared to the reference sequences in the NCBI GenBank database. Phylogenetic analysis showed that the two bacterial strains were *B. subtilis* with 99.73% similarity to NR 027552.1 (Supplementary Figure 4A) and *B. velezensis* with 99.93% similarity to NR 116240.1 (Supplementary Figure 4B).

Complete genome sequences of *B. subtilis* and *B. velezensis* have been submitted to the NCBI database, with the accession numbers PRJNA1272791 and PRJNA1272786, respectively. Bacterial genome maps of *B. subtilis* (Supplementary Figure 4C) and *B. velezensis* (Supplementary Figure 4D) were constructed through whole genome sequencing. The genome circle map has six circles. From outside to inside, they are: the first circle, genome coordinates. The second and third circles represent genes on the positive and negative strands of the genome, respectively, with COG classification indicated by color. The fourth circle shows rRNA and tRNA on the genomic sequence, where rRNA is blue and tRNA is red. The fifth circle displays the GC content profile of the genomic sequence. The sixth circle shows the GC skew curves of the genomic sequence, where green indicates a higher G content than C content and purple indicates a lower G content than C content.

## 4 Discussion

### 4.1 Treatment with microbial agents effectively regulate the physicochemical properties of rhizosphere soil in diseased peach trees

We conducted a detailed investigation into how different treatments affect soil physicochemical properties. *Bacillus* can reduce soil alkalinity through mechanisms such as metabolic activity or the secretion of organic acids (Yadav et al., 2024). In our study, the combined treatment with *B. subtilis* and a high concentration of GO (1,000 mg/L) (SH group) had the most significant effect on reducing the soil pH. The pH values of Group V and Group S were significantly lower than those of CK2 (*p* < 0.05) and close to the weakly basic range of the CK1 group. The OC content of Group V was the highest (26.43 g/kg) among all the treatment groups. In contrast, the OC contents of the remaining treatment groups were low, especially those of the SL group and the VH group, which may be related to the inhibition of microbial activity caused by the addition of GO (Vu et al., 2025). The Ca, Zn, and Mg contents were significantly higher in the CK2 group compared to the other groups. This may be due to the secretion of pathogenic substances, which severely damages the peach tree roots, leading to a reduced nutrient absorption rate and causing nutrients to accumulate around the roots (Chen et al., 2025). Althoughthe soil showed significantly lower levels of Ca, Zn, and Mg after treatment with *B. velezensis* (Group V) or *B. subtilis* (Group S) (*p* < 0.05). As plant rhizosphere growth-promoting bacteria, these two *Bacillus* species may have enhanced the root function of diseased peach trees, promoting the absorption and transport of nutrients by the plants, thereby depleting these elements in the rhizosphere soil (Xie et al., 2025). These beneficial microorganisms produce a variety of plant growth regulators and bioactive molecules, thereby increasing plant protection against stress (Masmoudi et al., 2019). In addition, after treatment with GO combined with microorganisms (VL, VH, SL, and SH groups), the soil zinc content was close to the level of the healthy peach tree CK1 group. *Bacillus* is a common plant growth-promoting rhizobacterium, and most of its members have the abilities to solubilize phosphorus, fix nitrogen, and strongly resist plant diseases (Huang et al., 2022). In our study, the TN, TP, and TK contents in the CK2 group were significantly higher than those in the other groups (*p* < 0.05), while the contents of all three in the V and S groups were relatively low, especially in the V treatment, which reduced the AP and AK contents by 80.2 and 51.4% compared to CK2, respectively. This is conducive to restoring the soil of diseased peach trees toward a normal state. GO had significant adverse effects on soil nitrogen cycle-related enzymes. Even at low concentrations of GO, it was able to inhibit the hydrolysis of urea and slow the nitrogen cycle in the soil (Fang et al., 2022). This could be the reason for the decrease in TN and AN content in the L group. However, in the SH group, the contents of AP and AK significantly increased. Studies have shown that *Bacillus* mainly enhances the bioavailability of phosphate and potassium in the soil by producing organic acids and secreting specific enzymes (Iqbal et al., 2024; Vitorino et al., 2024). *Bacillus* can improve the soil’s water retention capacity and promote the absorption of nutrients by plants (Ren et al., 2025). Compared with CK2 treatment, the introduction of Group V microbial mixtures reduced the contents of AN, AP, and AK by 32.9, 80.2, and 51.4%, respectively, bringing these values closer to those of the healthy peach tree CK1 group.

Results from the physicochemical study of the rhizosphere soil indicated that the rhizosphere of diseased peach trees (CK2) exhibited noticeable alkalization and excessive accumulation of salt ions (Ca, Zn, Mg). Treatment with a single microbial agent could improve soil conditions towards those of healthy controls (CK1) by lowering pH and promoting element absorption or transformation. When GO (L group) is used alone, soil TN and AN are inhibited. However, when GO is used in combination with *Bacillus*, depending on the concentration and strain, antagonistic or synergistic effects may occur. For example, the contents of AP and AK observed in the SH group were higher compared to SL, while the AP and AK levels in the VL group tended to approach normal levels. Overall, treatments using bacterial mixtures are more beneficial for restoring the physicochemical properties of the rhizosphere soil of diseased peach trees and improving the soil environment. In general, treatment with a bacterial mixture was more helpful in restoring the physicochemical properties of diseased peach rhizosphere soil and improving the soil environment.

### 4.2 Treatment with microbial agents modulate and reshape the microbial community to promote its functions toward a healthy state

Plant, soil and microbial communities are closely linked, and all three interact and influence each other (Li et al., 2023; Zahoor et al., 2024). Studies have revealed that applying microbial organic fertilizer can boost soil nutrient content, enhance beneficial microorganisms, and decrease harmful microorganisms (Pu et al., 2022). *Bacillus* spp. can increase the diversity of bacterial communities and affect the activities of various enzymes (Xia et al., 2025; Ahmed et al., 2022). In this study, the alpha diversity indices of root endophytic bacteria and rhizosphere fungi increased with the addition of *Bacillus* spp. inoculum. Higher microbial diversity is more conducive to maintaining soil health, promoting plant growth, and coexisting with microbial communities (Huang et al., 2025). Applying GO can reduce the abundance of microorganisms in the soil (Peña-Álvarez et al., 2024). This study found that, compared with the V group and S group, low-concentration GO treatment (L group) reduced the alpha diversity of root endophytic bacteria and soil fungi (Shannon and Simpson indices), which is consistent with previous research results (Wu et al., 2024; Du et al., 2020). This may be due to the direct physicochemical toxicity of low-dose GO to certain sensitive microorganisms, which disrupts the original community structure (Xie et al., 2020). Interestingly, the diversity index of endophytic bacteria in the high-concentration GO treatment group was higher than that in the low-concentration group. This may be because GO particles are more prone to aggregation at high concentrations, reducing their direct biological toxicity (Forstner et al., 2019). Although all mixed treatments consistently increased the Shannon index of the indigenous bacterial community (compared to CK2), their effects on soil fungal and bacterial diversity varied. For example, the application of the SL group caused a decrease in the Simpson index of soil bacteria, while the Simpson index of soil fungi increased (compared to CK2). In the SH group, the Simpson index of soil bacteria was the highest among all groups, while the Simpson index of soil fungi was the lowest among all groups. In general, a bacterial mixture or a high concentration of GO alone was more helpful in altering the microbial diversity of peach trees.

Among the endophytic bacterial communities, Cyanobacteria presented the highest abundance in the CK2 group. As photoautotrophs, Cyanobacteria not only have the ability to fix biological nitrogen, but also affect the stability of microbial communities by producing extracellular polymeric substances (Nawaz et al., 2024; Xu et al., 2025). The plant may respond to disease stress by recruiting diazotrophic bacteria in order to maintain its growth needs (Zhang et al., 2022). Compared with CK2, the abundance of Proteobacteria, Actinobacteriota, and *Streptomyces* increased in all treatment groups, while the abundance of *Pseudomonas* increased the most in the SH group. These groups of bacteria can promote plant growth and protect plants from damage by inhibiting the growth and reproduction of pathogenic bacteria (Cao et al., 2021). Furthermore, the use of microbial agents altered the bacterial community diversity in the rhizosphere soil and positively impacted the structure of rhizosphere bacteria (Lai et al., 2024). However, the results of the present study revealed that the addition of a bacterial mixture did not significantly change the abundance of *Bacillus* spp. in the soil, which was consistent with previous study results (Pan et al., 2025). This may be due to competition between local microbial communities and introduced beneficial microbes, thereby increasing the difficulty of colonization by nonnative microbes (Xue et al., 2019). Compared with that in CK2, the abundance of Proteobacteria decreased in the different treatment groups, whereas the abundances of Acidobacteria and Crenarchaeota increased. Acidobacteria is one of the main flora in soil and plays an important role in nutrient cycling, especially in the sulfur and nitrogen cycles (Gonçalves et al., 2024). Crenarchaeota has strong environmental adaptability and is important for maintaining material cycling and energy flow in the ecosystem (Gu et al., 2013). After treatment, the abundance of *Sphingomonas* in the soil bacteria increased in all groups, and the abundance was higher than that of CK1. These bacteria can support plant growth and enhance productivity (Pecourt et al., 2025). Compared to other groups, *RB41* is most abundant in the L group. *RB41* is widely distributed in soil and is often closely associated with organic matter decomposition and the carbon cycle (Stone et al., 2021). In summary, the research results indicate that the applied treatments, especially the *Bacillus*-GO combination, reshaped the bacterial communities in the rhizosphere and roots of diseased peach trees by enriching beneficial bacteria associated with nutrient cycling and plant growth promotion. Although the introduced *Bacillus* did not dominate the soil, it appears to play a key regulatory role, guiding the microbial community toward a functional state more similar to that of healthy plants (CK1).

The changes in the soil fungal communities varied among the different treatment groups. Compared with that in the CK2 group, the abundance of *Arxiella* increased in Groups V and S after bacterial mixture treatment. In Group L, the abundance of *Penicillium* increased significantly. *Penicillium* can decompose complex organic matter in soil, promote nutrient cycling, and produce antibiotics to inhibit the growth of pathogens (Bahram et al., 2018). The results of the present study revealed that plants respond to disease stress and maintain their growth needs by regulating the microbial community structure in the rhizosphere, especially in regulating the abundance of beneficial bacteria such as nitrogen-fixing bacteria. Previous studies have shown similar results (Hadian et al., 2025; Mataranyika et al., 2024). Although the application of microbial inoculants did not significantly change the abundance of *Bacillus* in the soil, it optimized the functional diversity of bacterial and fungal communities and enhanced soil nutrient cycling.

Correlation analysis revealed the relationships between soil environmental factors and predominant bacterial taxa. In general, changes in soil physicochemical properties had a more significant impact on rhizosphere bacteria, and most physicochemical parameters were positively correlated with bacterial abundance. Yan et al. (2025) reported that the application of biochar-based fertilizers improved key soil properties (such as organic carbon and total nitrogen), thereby increasing the richness and diversity of soil bacterial communities, and ultimately promoting the growth and yield of tobacco (Yan et al., 2025). In contrast, the abundance of endophytic bacteria and most physicochemical indicators was negatively correlated, while only a few physicochemical indicators were significantly correlated with the abundance of soil fungi. The functions of the microbial community also differ between different groups. The DNA repair and response regulators of the endophytic bacteria were significantly increased in CK2. The enhancement of these functions not only helps the survival and reproduction of the bacteria themselves but also has a positive effect on the host plants, improvement in stress tolerance and growth performance of plants (Kravchenko et al., 2020). This directly reflects the self-regulation of the microbiota to survive in the rhizosphere adversity caused by disease stress. The same was true among the rhizosphere bacteria. The CK2 group presented an increased environmental response to improve plant stress tolerance. After different treatments, the functions of bacteria were more closely related to those of the CK1 group in healthy plants. This was especially the case for the VH group and L group. The L-group enhanced Nucleoside-diphosphate-sugar epimerase is involved in the conversion of sugar nucleotides, which is a prerequisite for the biosynthesis of various sugars. NAD(P)-dependent dehydrogenases are directly involved in the catabolism of organic compounds such as sugars and alcohols and in energy production. Both are beneficial for plant growth. The functional composition and trophic patterns of the fungi significantly differed among the different treatment groups. CK2 had a relatively high proportion of pathotrophs, indicating that pathogenic fungi may be dominant in diseased soil. Pathotrophs saprotrophs symbiotrophs and saprotrophs pathotrophs symbiotrophs were the main players in Groups L and VH, respectively, suggesting that symbiotrophs play important roles in the stability of fungal communities in rhizosphere soil (Zhou et al., 2024). These findings suggest that these treatments may shift the fungal community toward a more balanced ecological function, possibly helping to restore soil health.

## 5 Conclusion

In this study, the effects of the *Bacillus* spp. inoculum agent, graphene oxide (GO), and its composite treatments on the physicochemical properties and microbial communities of diseased peach rhizosphere soil were analyzed. Different treatments relieved the stress caused by the disease on peach trees to a certain extent. In particular, the bacterial mixture increased the diversity of microorganisms in the peach root system, and the physicochemical properties of the rhizosphere soil and the functions of the microbial communities tended to be normal. These changes may directly affect nutrient cycling in the soil, providing a new environment for peach tree growth and jointly promoting its development through alterations in endophytic bacteria. The results of the GO treatment differed due to their different concentrations. A high concentration of GO enhanced microbial diversity, whereas a low concentration of GO reduced microbial diversity. In the composite treatment, the synergy of *B. velezensis* and GO was particularly prominent. Through the coordination of the optimization of soil physicochemical parameters and the reorganization of microbial functional modules, a more complete rhizosphere microecological network was constructed. This study not only elucidated the mechanism of action of biofungicide in mitigating soil-borne diseases of peach trees from the dimension of “element cycling–microbe interaction,” but it also innovatively proposed a composite regulation strategy of GO–microbes. This strategy provided quantifiable contributions for the ecological restoration of the peach rhizosphere. The theoretical framework has deepened our understanding of the plant–microbe-soil interaction network.

## Data Availability

The original contributions presented in the study are publicly available. This data can be found here: https://www.ncbi.nlm.nih.gov/, accession numbers PRJNA1394517 and PRJNA1394521.

## Footnotes

1
https://www.arb-silva.de/

2
https://unite.ut.ee/
