# Supplementary material for: Synergistic effects of Bacillus spp. and graphene oxide on nutrient cycling and soil health in peach tree rhizospheres
Source: Front Microbiol. 2026 Feb 13;16:1712181. doi: 10.3389/fmicb.2025.1712181 (PMC12946128; doi:10.3389/fmicb.2025.1712181)
Supplement: Supplementary file 1 [file Image_1.pdf]

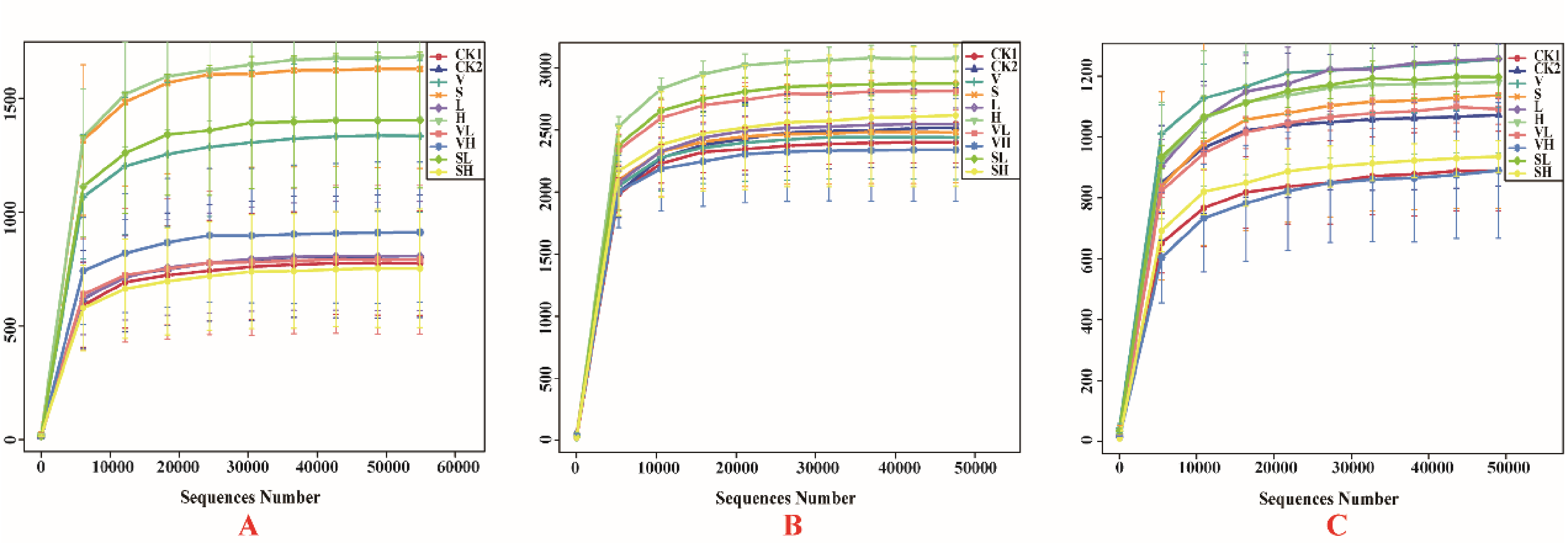

Figure S 1 Dilution curves for root endophytes (A), inter-root soil bacteria (B) and inter-root soil fungi (C).

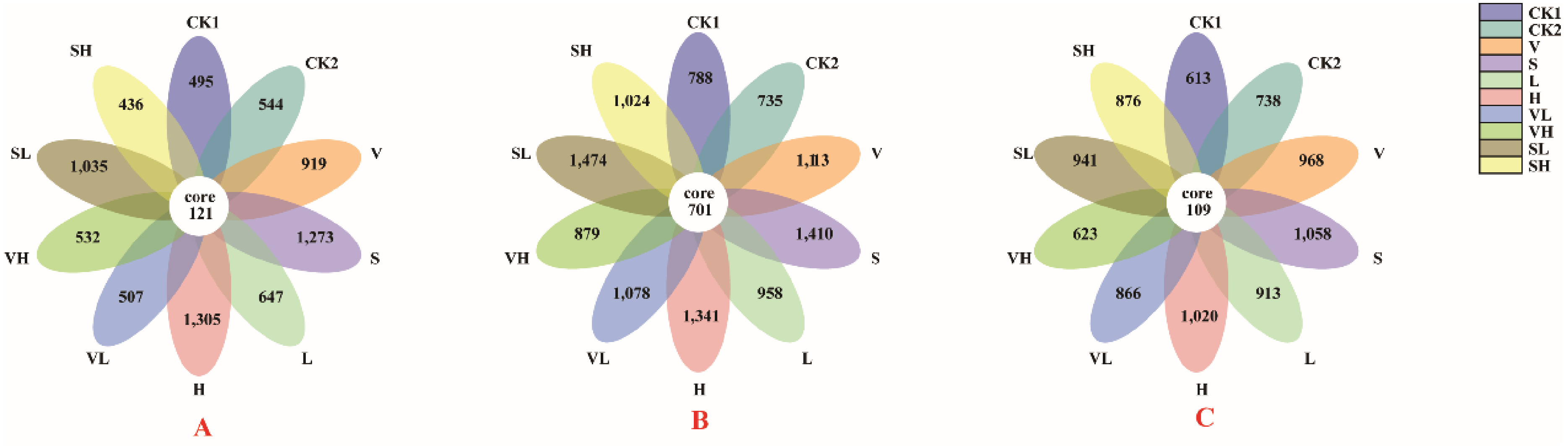

Figure S 2 OTU petalograms of root endophytes (A), inter-root soil bacteria (B) and inter-root soil fungi (C).

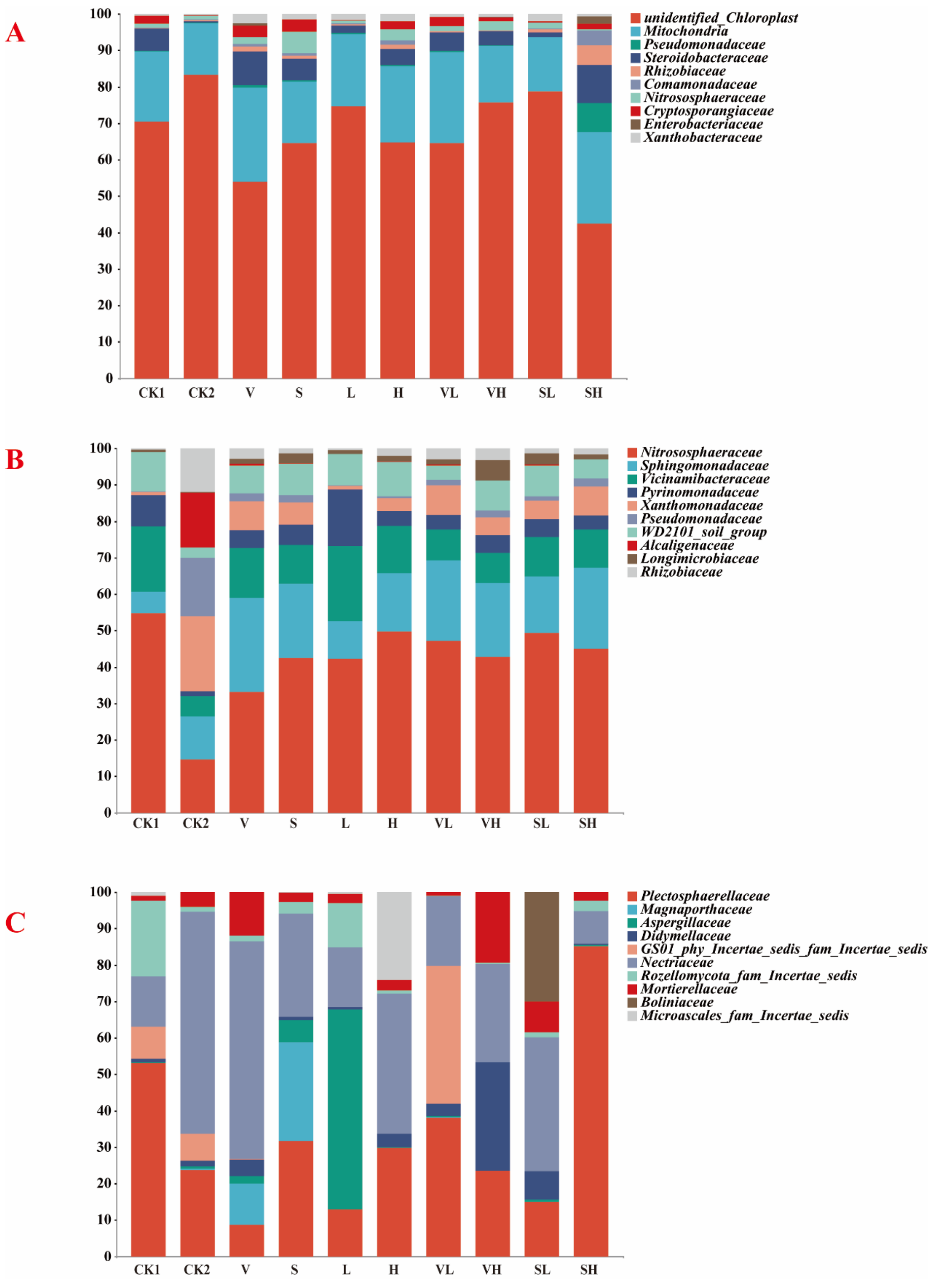

Figure S 3 Family-level relative abundance of top 10 taxa of root endophytes (A), inter-root soil bacteria (B) and inter-root soil fungi (C).

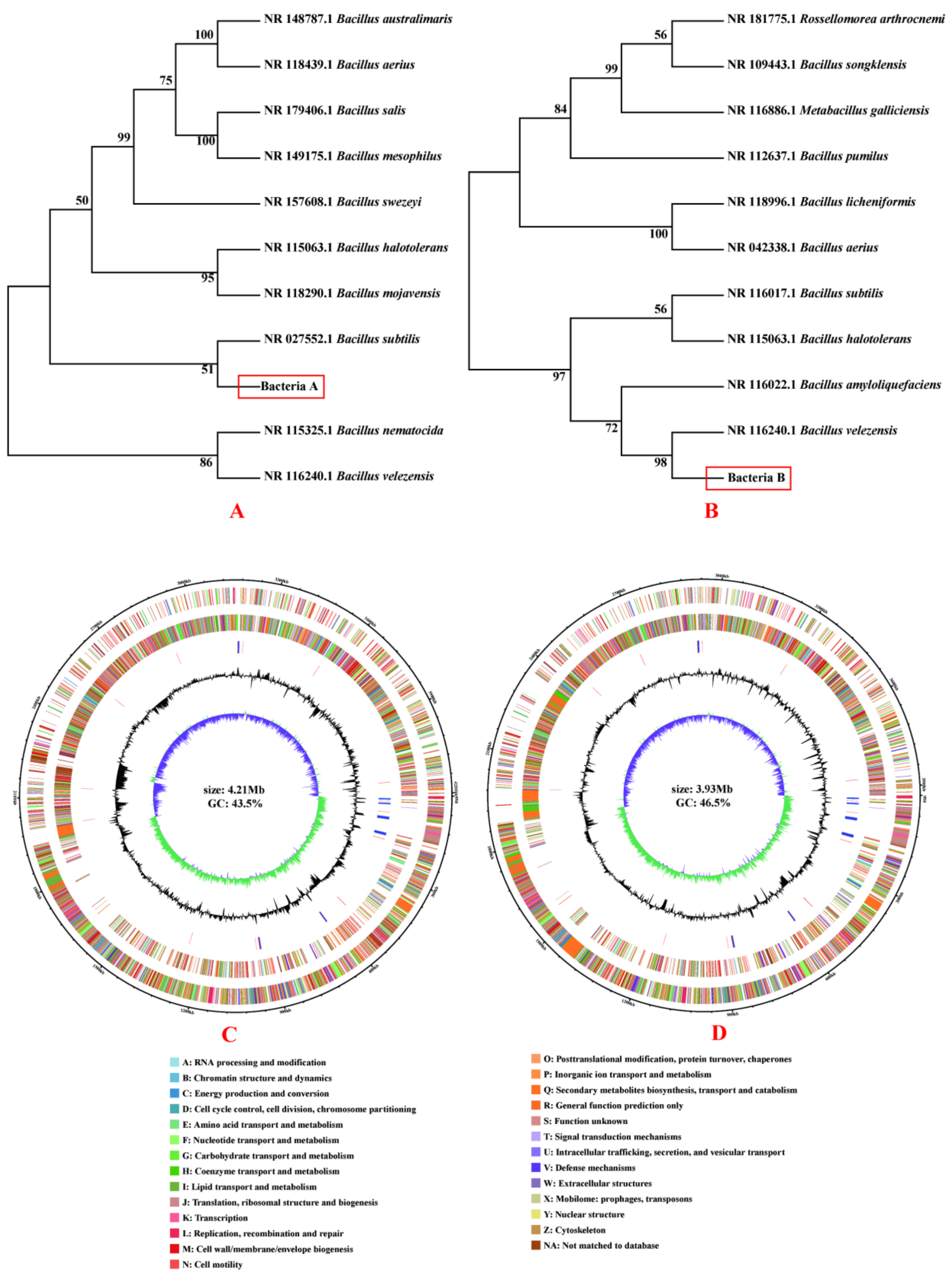

Figure S 4 Identification diagram of the two strains of Bacillus used in the experiment. (A)Phylogenetic tree of *Bacillus subtilis*, (B) Circos map of the *Bacillus subtilis* genome, (C) Phylogenetic tree of *Bacillus velezensis*, (D) Circos map of the *Bacillus velezensis* genome.
